# A fatal case of Munchausen syndrome by proxy in the father–daughter dyad

**DOI:** 10.1007/s12024-026-01231-8

**Published:** 2026-03-24

**Authors:** Federica Amirante, Roberto Catanesi, Biagio Solarino, Felice Carabellese, Anna Margari

**Affiliations:** 1 Section of Legal Medicine, Interdisciplinary Department of Medicine, University of Bari Aldo Moro, Piazza Giulio Cesare 11, Bari, Italy; 2 Section of Criminology and Forensic Psychiatry, Interdisciplinary Department of Medicine, University of Bari Aldo Moro, Piazza Giulio Cesare 11, Bari, Italy

**Keywords:** Munchausen syndrome by proxy, Pediatric abuse, Infanticide, Asphyxia, Forensic pathology

## Abstract

Factitious Disorder Imposed on Another (FDIA), formerly known as Munchausen Syndrome by Proxy, is a rare form of abuse where parents induce or fabricate symptoms in their child, exposing them to unnecessary and potentially harmful medical treatments. In over 90% of cases, the perpetrators are mothers. A father was put on trial for the murder of his three-month-old daughter and was eventually convicted of repeatedly inducing respiratory crises, ultimately causing her death. Investigations began after multiple visits to the Emergency Department for respiratory episodes reported by the father but not confirmed by physicians: the attending paediatrician first hypothesized FDIA. A medico-legal autopsy excluded diseases justifying prolonged hospitalization or independently causing death; the only relevant post-mortem findings were massive pulmonary congestion with minute subpleural petechiae and hemosiderin-laden intra-alveolar macrophages, indicating nonspecific acute respiratory failure. Forensic psychiatric evaluation of the father revealed a rigid personality, excessive conformity and interpersonal immaturity. Publicly, the father appeared caring and deeply involved with his daughter. He exhibited a propensity to develop somatic symptoms and a tendency towards reality distortion. These traits aligned with FDIA, later confirmed by judicial reconstruction of events. The multidisciplinary approach led to the establishment of the judicial truth: a case of paternal infanticide by asphyxiation. In Italy, FDIA is not a mental illness excluding criminal responsibility, though the diagnosis may influence sentencing. The first-instance judgment classified the act as preterintentional homicide, considering Munchausen by proxy. The final verdict, however, convicted the father of intentional homicide. This case highlights the intrinsic diagnostic dilemma at the clinical–forensic interface, where psychiatric assessment and judicial interpretation may align or diverge, underscoring the need for caution in translating psychiatric diagnoses into legal conclusions.

## Introduction

Factitious Disorder Imposed on Another (FDIA), formerly known as Munchausen Syndrome by Proxy (MSBP), was first described by Meadow in 1977. It refers to parents who deliberately fabricate or induce symptoms, thereby exposing their children to numerous unnecessary and potentially harmful diagnostic or therapeutic procedures [1].

The name derives from Baron von Münchausen, an 18th-century German mercenary renowned for exaggerated tales of his military exploits, an emblem of reality distortion [2]. In the literature, the condition is sometimes labelled “Polle syndrome” after a legend alleging the suspicious death of the baron’s son [3, 4].

Although the eponym is male, epidemiological data show a clear female predominance. In 92%–100% of reported cases, the perpetrator is the child’s mother, and 76.5%–100% are biological mothers; active paternal involvement is exceptionally rare [5–7]. Scientific observations of male perpetrators remain scant; Meadow’s 1998 series of 15 fathers is one of the few substantial contributions [8].

Available incidence figures almost certainly underestimate the true prevalence of the condition, as underreporting is common and directly affects epidemiological estimates, given the deceptive nature of the perpetrator’s behaviour and the heterogeneity and ambiguity of clinical presentations [9]. Moreover, abusers frequently present as attentive and devoted caregivers, further complicating recognition by healthcare professionals and hindering timely identification of the abuse [10]. Rosenberg’s 1987 work was pivotal in outlining the core features of MSBP (Appendix 1) (2). The DSM-5-TR classifies the syndrome as “Factitious Disorder Imposed on Another” (FDIA) (Appendix 2) [11].

Munchausen syndrome by proxy falls within the broader category of Medical Child Abuse (MCA), in which the young victim is subjected to unnecessary, harmful, or potentially harmful medical care initiated by a caregiver [7]. Importantly, only a minority of caregivers who induce symptoms meet full clinical criteria for classic MSBP [12].

Mortality in Fabricated and Induced Illness is estimated at 9%–22% [13]. Suffocation-induced apnoea, a specific and dangerous MSBP subset, together with poisoning episodes, accounts for nearly all fatal cases. Induced suffocation is also a key differential diagnosis in suspected Sudden Infant Death Syndrome (SIDS), having been implicated in deaths initially classified as sudden [14, 15]. Craft and Hall estimate that up to 10% of crib deaths may be due to deliberate caregiver suffocation, an extreme manifestation of MCA [12].

Given its clinical and legal complexity, FDIA demands a multidisciplinary approach [16]. When death is suspected, an autopsy is essential for accurate determination of cause and manner of death, supplemented by targeted toxicology of blood, urine, gastric contents, and vitreous humour to detect toxic agents or non-indicated drugs. The absence of standard forensic protocols for Fatal FDIA, however, hampers definitive conclusions, partly because of heterogeneous clinical manifestations [17].

As with other forms of child abuse, proof often relies on indirect or circumstantial evidence; the abusive act is seldom witnessed [18]. Retrospective review of medical records can be crucial, especially when discrepancies arise between the caregiver’s account and objective findings. Symptoms that appear only in the caregiver’s presence and resolve in their absence must be meticulously documented, as they may constitute key evidence [19].

Psychiatric classifications such as FDIA are designed for clinical, not legal, purposes. Misuse of these labels in court can lead to serious misinterpretation: a diagnosis may be invoked either to excuse wrongdoing or, conversely, as “proof” of abuse that never occurred [20].

Robust clinical-forensic management, therefore, requires comprehensive information gathering, rigorous medico-legal evaluation, and cautious application of psychiatric diagnoses in legal contexts.

We present a rare fatal case of paternal FDIA culminating in a criminal conviction for the intentional homicide of a newborn daughter. Owing to the scarcity and fragmentation of sources describing male perpetrators, this case offers valuable insights. It underscores the technical challenges of evidence collection and highlights the pivotal role of multidisciplinary collaboration, particularly medico-legal and psychopathological assessments, in establishing judicial truth and determining both the legal classification of the act and the sentence.

Appendix 1 – Munchausen Syndrome By Proxy Definition (Rosenberg D, Child Abuse & Neglect, 1987): (1) Illness in a child which is simulated (faked) and/or produced by a parent or someone who is in loco parentis; and (2) Presentation of the child for medical assessment and care, usually persistently, often resulting in multiple medical procedures; and (3) Denial of knowledge by the perpetrator as to the aetiology of the child’s illness; and (4) Acute symptoms and signs of the child abate when the child is separated from the perpetrator.

Appendix 2 – Factitious Disorder Imposed on Another Diagnostic Criteria (DSM 5-TR): (1) Falsification of physical or psychological signs or symptoms, or induction of injury or disease, in another, associated with identified deception. (2) The individual presents another individual (victim) to others as ill, impaired, or injured. (3) The deceptive behaviour is evident even in the absence of obvious external rewards. (4) The behaviour is not better explained by another mental disorder, such as delusional disorder or another psychotic disorder.

## Case description

### The neonate’s clinical history

This case concerns a rare and striking judicial proceeding involving a father charged with the homicide of his three-month-old daughter. During the investigative phase, the public prosecutor commissioned a series of expert evaluations, including a full autopsy, a review of the infant’s medical records and a forensic psychiatric assessment of the accused.

Analysis of the medical documentation revealed that the infant, born following an uneventful pregnancy and without obstetric or perinatal complications, was taken to the Emergency Department on six separate occasions during her short life. These visits led to five hospital admissions, amounting to a total of 73 days of hospitalization. Table 1 summarises the duration and circumstances of each admission.

**Table 1:** Summary of the neonate’s clinical history

Hospital admission / ED visit	Length of stay (days)	Description
1st Admission	12	The father reported finding the infant asleep in her crib, with traces of blood emerging from her nose and mouth. The infant was admitted to the Neonatal Intensive Care Unit, where a diagnosis of bronchiolitis was made upon discharge, although no causative agent could be identified.
2nd Admission	7	In a subsequent episode, reported by one of the parents, though it remains unclear which, an incident of haemoptysis was described. According to the account, the infant experienced a feeding-related regurgitation episode, followed by oral bleeding, fever, and a brief episode of cyanosis. She was admitted to the Pediatric Unit, where she later suffered a severe desaturation episode that culminated in cardiac arrest.
3rd Admission	29	Hospitalization continued in the NICU, reportedly due to status epilepticus and cardiorespiratory failure in a patient with intermittent gastric volvulus.
4th Admission	23	The father later reported a prolonged episode of apnoea and cyanosis, lasting more than five minutes, which he claimed resolved only after external stimulation. The infant was admitted to the Neonatal Unit and discharged with a diagnosis of rhinovirus-induced rhinitis, confirmed by oropharyngeal swab.
ED Visit (no admission)	-	The father reported an episode of respiratory distress followed by inconsolable crying, which was assessed as non-critical.
5th Admission (Final)	2	During an outpatient follow-up visit with the general paediatrician, extrasystoles were detected on auscultation. The parents also reported episodes of dyspnea and oxygen desaturation. The infant was admitted once again to the Pediatric Unit, where, after two days of hospitalization, she was pronounced dead.

Across these hospital stays, a common pattern emerged: the recurrence of unexplained nonspecific respiratory crises, almost exclusively reported by the infant’s father to medical personnel. These episodes, typically characterized by desaturation and apnoea, were managed through oxygen therapy. One particularly severe event resulted in cardiorespiratory arrest, prompting the medical team to transfer the child to the Neonatal Intensive Care Unit (NICU).

To clarify any underlying organic cause, extensive clinical and instrumental investigations were performed, all of which yielded essentially negative results. Throughout the various hospitalizations, the following tests were conducted: 8 echocardiograms, 7 electrocardiograms (ECG), 3 cranial CT scans, 11 chest X-rays, 5 pulmonary ultrasounds, 3 abdominal X-rays, 1 chest CT scan, 10 electroencephalograms (EEG), 2 Holter ECG monitorings, multiple bacterial cultures, and a comprehensive metabolic screening. None of these investigations revealed any pathological condition that could explain the reported respiratory symptoms. The only notable findings were a clinical diagnosis of bronchiolitis during the first admission and a critical desaturation episode with cardiac arrest during the second.

Based on the combination of recurrent, caregiver-reported acute events and the absence of objective diagnostic findings despite extensive investigations, during the third hospitalization, the head of the NICU filed a report with judicial authorities, raising suspicion of child abuse consistent with FDIA.

On the day of death, the infant experienced a desaturation episode with apnea and cyanosis around 12:30 p.m., which resolved with oxygen therapy. An ECG Holter monitor was applied at 1:00 p.m., showing normal tracings until 11:47 p.m., when sinus bradycardia appeared. The last feeding reportedly took place at about 11:00 p.m., and a blood gas analysis at 11:40 p.m. revealed normal respiratory parameters. At 11:55 p.m., the father alerted the on-duty nurse due to a sudden deterioration. The infant was found in cardiac arrest, cyanotic, with milk oozing from the nose and mouth; approximately 100 ml of milky material was aspirated from the airways. Despite immediate resuscitation attempts and transfer to the intensive care unit, death was declared at 1:10 a.m.

Considering the previous report of suspected abuse, the judicial authorities launched a formal investigation.

### Forensic assessments – post-mortem examination of the infant

Before the autopsy, radiological investigations were conducted on the cadaver in accordance with established protocols for cases of sudden infant death syndrome (SIDS) [21]. A full-body CT scan ruled out the presence of bone trauma throughout the body. No evidence of traumatic injury or visceral pathology was found.

Toxicological screening, performed in accordance with international recommendations [17], yielded negative results for psychotropic medications and drugs of abuse.

The external examination of the body confirmed normal weight gain and the absence of relevant abrasions or bruising. Livor mortis was prominent in dependent and posterior areas of the body, consistent with the decubitus position of the cadaver.

The autopsy revealed that all parenchymal organs were diffusely affected by vascular congestion. In the respiratory system, the airways were patent, but the lungs appeared heavy and firm on sectioning, with subpleural petechiae at the bases. The coronal section of the heart showed a whitish area in the myocardium, located in the proximal third of the interventricular septum, extending inferiorly below the valvular plane into the membranous portion for approximately 0.6 cm longitudinally and 0.5 cm transversely across the septum. The esophagus was empty, while the stomach contained stratified milky material.

Histopathological analysis provided further detail. Microscopic examination of lung fragments revealed marked congestion, diffuse hypoaeration, and intra-alveolar desquamation. The latter consisted of pneumocytes, bronchial epithelial cells, and foamy macrophages, some of which contained hemosiderin granules. Although these findings are largely non-specific and may be observed in a variety of terminal conditions, including different asphyxial mechanisms, thereby posing significant interpretative challenges when considered in isolation, they are nonetheless consistent with acute respiratory failure and generalized multiorgan congestion. The absence of exogenous toxic substances supported this interpretation. Moreover, no evidence of diffuse inflammatory processes (such as bronchopneumonia, bronchiolitis, or alveolitis) was found, in line with the normal blood gas values obtained shortly before death. The alveolar desquamation and reduced lung aeration may have been partially influenced by prolonged resuscitative manoeuvres.

Histological analysis of the whitish area observed in the interventricular septum revealed a hypocellular fibrotic zone. Within this fibrotic tissue, small hyperbasophilic calcium deposits were identified, along with foci of histiocytic-macrophagic cells and occasional lymphocytes, as confirmed by immunohistochemical staining. This finding is indicative of a prior inflammatory process, compatible with myocarditis, although there was no active inflammation at the time of death. Based on morphological features, the onset of this fibrotic process was estimated to have occurred approximately 8–10 weeks prior to death [22]. It was hypothesized that this inflammatory process could have been related to the first hospitalization, during which a viral bronchiolitis was diagnosed, possibly also responsible for myocardial involvement [23].

The integrated analysis of objective findings and clinical documentation led to the conclusion that the infant’s death was due to a non-specific acute respiratory failure, in the absence of any preexisting organic pathology that alone could explain the fatal outcome.

While the hypothesis of an accidental aspiration event (e.g., post-feeding regurgitation) is theoretically possible in infancy, it was deemed incompatible with clinical and post-mortem evidence. A fatal asphyxial event would require significant inhalation of gastric contents, such as massive vomiting, not just mild reflux. The blood gas analysis performed shortly before death showed normal respiratory parameters, excluding any ongoing respiratory insufficiency. This suggests that the fatal event occurred within a very short time frame thereafter, making it highly unlikely that an asphyxial mechanism of such magnitude occurred without classic signs of respiratory distress (tachypnea, progressive desaturation, cyanosis, agitation, etc.).

Although septal fibrosis may predispose the patient to serious arrhythmias, multiple cardiological investigations (echocardiograms, ECGs, Holter monitoring) yielded negative results for rhythm or conduction abnormalities. The final clinical picture was dominated by sinus bradycardia, a finding more frequently associated with acute hypoxia of respiratory origin. No evidence emerged to support a direct causal role of the interventricular septal fibrosis in the death, though a contributory effect in the context of acute respiratory failure was hypothesized [24].

No respiratory pathology was identified that could coherently explain both the death and the repeated hospital admissions. The presence of hemosiderin-laden macrophages in the lungs is a rare finding, but one that may be associated with acute asphyxial events, as documented in the literature [25]. During the first hospitalization, a likely viral bronchiolitis was diagnosed, with suspected myocardial involvement. However, an atypical feature was the occurrence of hemoptysis, reported by the parents, a symptom rarely associated with bronchiolitis and, in this case, not corroborated by objective or instrumental findings affecting the lower airways. The most alarming clinical manifestations systematically occurred at home and were never objectively documented in the hospital, where the child’s condition was consistently good enough to allow for discharge.

### Forensic assessments – psychopathological evaluation of the accused father

The father is a 29-year-old male with a normative developmental history and a high school diploma in accounting. Despite a standard social background, his academic years were marked by interpersonal conflict with authority figures, and his adult life is characterized by professional instability and a lack of specialized employment. The parental relationship was established five years prior to the child’s birth.

From a clinical standpoint, he presented a complex psychological profile, characterized by a recurrent tendency to distort facts in a manner functional to his immediate interests. His narratives regarding social and family relationships were often unrealistic, constructed and marked by manipulative attitudes and a lack of moral restraint. According to close relatives, including his mother and partner, he frequently lied or feigned illness, particularly in stressful situations or when facing consequences for his actions. Of note, a past episode of a feigned suicide was reported. These behaviours suggest a longstanding pattern of dissimulation and attention-seeking. In the five years preceding his daughter’s birth, he made 27 emergency department visits, predominantly for vague somatic complaints, non-specific pain, functional disturbances (including conversion symptoms and pseudoneurological presentations), self-injurious behaviour, and alleged physical assaults.

Following the events involving his daughter and the ensuing criminal investigation, he exhibited no significant emotional distress; on the contrary, he appeared somewhat pleased to be at the centre of attention. Cognitive impairments and psychotic-spectrum disorders, including delusional falsification, were clinically excluded. Instead, personality assessment revealed prominent histrionic traits rooted in a narcissistic core, driving a self-centred and highly subjective interpretation of events and interpersonal dynamics.

A comprehensive psychodiagnostic evaluation was conducted using the MMPI-2, Rorschach Test and the Psychopathy Checklist-Revised (PCL-R). The subject obtained a PCL-R T score of 45, which is below the established threshold indicative of psychopathy. The profile is characterized by a marked predominance of interpersonal/affective features (Factor 1 T score = 57, Factor 2 T score = 40). The results were mutually consistent and aligned with clinical observations, supporting a diagnosis of a personality disorder with predominant Cluster B features, specifically narcissistic and histrionic traits. This condition was not deemed severe enough to compromise his criminal responsibility (i.e., capacity to understand and to will). Additionally, based on the available clinical history and documented behavioural patterns, a diagnosis of Somatic Symptom Disorder was established.

In considering the hypothesis of FDIA, which is the updated DSM-5-TR term for identifying MSBP, clinical assessment revealed psychological features consistent with profiles described in the literature, including somatization tendencies and reality distortion, particularly through exaggerated caregiving involvement. However, per DSM-5-TR criteria, a diagnosis of FDIA requires the intentional falsification of physical or psychological signs or symptoms, which cannot be inferred solely from psychiatric evaluation, as no definitive clinical markers exist. Thus, the psychiatric formulation in such cases must remain subsidiary to, and contingent upon, forensic and procedural findings: only after the deliberate production of symptoms in the child is formally established within the judicial process - through cross-examination of the evidence and legal attribution of responsibility - can the coherence between the subject’s psychological profile and the offense serve as an additional element of consistency. In the present case, the subject’s psychological makeup and behavioural history delineated a profile markedly aligned with clinical patterns recurrently documented in cases of FDIA. This configuration, while not diagnostic in itself, provided a coherent and substantiated framework which may support a formal diagnostic confirmation, contingent upon the judicial establishment of deliberate symptom fabrication or induction.

### Forensic assessment implications for the court’s ruling

A comprehensive multidisciplinary forensic assessment integrating pathological, contextual, and forensic psychiatric evidences enabled the court to reconstruct event dynamics and reach a judicial determination of paternal infanticide by asphyxiation. In the initial trial, the defense advanced a hypothesis of FDIA to explain the father’s conduct. In the Italian legal framework, FDIA does not constitute a mental disorder that excludes or reduces criminal responsibility. The psychiatric assessment recognized that the defendant was fully responsible.

Nonetheless, its recognition can influence the legal qualification of the offense and potentially the sentencing. In the first-instance judgment, the court acknowledged elements that classified the act as preterintentional homicide, reflecting an interpretation of diminished awareness or indirect intent. The final ruling requalified the conduct instead as intentional homicide, thereby attributing full criminal liability to the perpetrator.

## Discussion

This case represents a rare fatal instance of paternal FDIA, resulting in the intentional suffocation of a 3-month-old infant and subsequent conviction for homicide.

This case was examined through a dual forensic perspective, integrating the medico-legal autoptic evaluation of the victim with a forensic psychiatric assessment of the perpetrator. Such an approach allows for a comprehensive reconstruction of both the cause and mechanism of death and the underlying criminodynamic and psychopathological features of the offender. By addressing the case simultaneously from the standpoint of the deceased infant and that of the caregiver ultimately convicted of homicide, this report aims to highlight the added value of an interdisciplinary forensic framework in the investigation of fatal FDIA cases.

From the medico-legal autoptic perspective of the victim, a thorough diagnostic workup was conducted, in accordance with forensic protocols for sudden unexpected infant death (SUID), including suspected cases of SIDS [26]. This included full-body radiological imaging, complete autopsy, histopathological analysis, and comprehensive toxicological screening [17]. All investigations were performed in adherence to internationally accepted guidelines to exclude natural, accidental, or undetermined causes of death. Importantly, the integrated clinical, laboratory, and post-mortem findings excluded known causes of natural death in infancy, such as bronchiolitis, myocarditis with active inflammation, metabolic disorders, or inherited arrhythmias. The presence of intra-alveolar hemosiderin-laden macrophages, although non-specific, has been described in the context of acute asphyxial deaths and was interpreted here as a possible marker of repeated hypoxic events [27]. However, such findings, when considered in isolation, lack sufficient specificity to establish a definitive cause of death, highlighting the inherent limitations of post-mortem pathology in distinguishing between natural and non-natural asphyxial mechanisms. The diagnosis of SIDS requires not only the absence of a clear cause of death after thorough investigation but also the lack of suspicious circumstances. In this case, however, a pattern of recurrent hospitalizations with unexplained symptoms, the absence of any documented deterioration in hospital settings, and the eventual fatal event, despite appropriate care and surveillance, strongly argued against SIDS as a viable diagnosis. Thus, the systematic exclusion of natural causes, combined with the caregiver’s psychosocial and behavioural profile, pointed toward a hypothesis of asphyxial death by intentional suffocation. This underscores the critical importance of a rigorous differential diagnostic process in distinguishing natural from non-natural causes of death in infancy. Beyond its tragic outcome, the case illustrates the essential role of scientific hypothesis testing in forensic medicine: through methodical exclusion, the diagnostic process supported a violent aetiology, which was subsequently corroborated by circumstantial and investigative findings.

From the forensic psychiatric perspective of the perpetrator, paternal FDIA remains poorly documented in the literature, with Meadow’s 1998 series of 15 cases still serving as a primary reference [8].

The perpetrator’s age (29 years) fits within the range reported by Meadow, who described young male offenders, typically under 30. However, the victim was significantly younger than the mean age reported in the literature, and the abusive behaviour appears to have begun early in infancy and escalated rapidly, culminating in death shortly after the first formal suspicion of FDIA.

Symptomatically, the case mirrors Meadow’s findings, with recurrent caregiver-reported episodes of apnea and cyanosis lacking objective confirmation or organic explanation, and the father consistently presenting himself as the sole witness and first responder.

A particularly relevant aspect of this case is that the fatal event occurred during hospitalization, despite a prior report filed by the head of the neonatology unit. Unlike some previously reported cases in which abusive acts were directly observed or documented through surveillance, no direct visual evidence was available. This finding emphasises that hospitalization alone may not be sufficient as a protective measure once abuse is suspected.

From a psychiatric standpoint, the perpetrator exhibited Cluster B personality traits, predominantly narcissistic and histrionic, along with a diagnosis of somatic symptom disorder. Although no psychiatric diagnosis had been established at the time of the events, these findings are consistent with patterns reported in male FDIA perpetrators, who frequently display somatization and factitious behaviours. In this case, the cessation of the father’s repeated emergency department visits for unexplained symptoms following his daughter’s birth further supports a shift in the focus of symptom fabrication from the caregiver to the child.

Unlike some of Meadow’s cases, in which partial admissions were made, our subject never confessed. The legal process concluded with a conviction for intentional homicide, and psychiatric insights emerged solely within the forensic context. In this setting, the evaluation of a possible diagnosis of FDIA had to proceed with particular caution, given that such classifications are intended for clinical, not judicial, use [20]. Rather than serving as the basis for establishing the abusive act, the diagnosis was necessarily subordinated to the prior factual determination of wrongdoing, in line with the principles of a sound clinical-forensic approach.

From a medico-legal perspective, it is essential to stress that the mere compatibility between certain psychological traits and a specific criminal behaviour cannot itself serve as a sufficient diagnostic tool. Traits such as manipulativeness, attention-seeking behaviour, and reduced affective empathy are indeed frequently described in individuals with FDIA, yet they lack the specificity and diagnostic power required to establish the condition in the absence of clear, corroborated facts.

The FDIA label carries inherent risks, potentially leading to the juridification of a medical concept and, conversely, the medicalization of legal matters. Labelling a set of behaviours as a “syndrome” can foster a false sense of diagnostic certainty in court, obscuring the clinical nuance that a syndrome does not constitute a definitive diagnosis [28]. Accordingly, in legal proceedings concerning sentencing for individuals with FDIA who have committed serious offences, the focus should shift from the syndrome itself, given its evolving and sometimes debated diagnostic boundaries, to the underlying personality dysfunction. This approach ensures that judicial arguments address those aspects of an offender’s personality structure that directly influence culpability, rather than relying on a clinical condition that merely describes the criminal behaviour [29].

In our case, however, this conceptual ambiguity was overcome by the progression of events. The conviction for intentional homicide provided a robust factual foundation for retrospective psychiatric evaluation. It was within this framework that the diagnosis of FDIA was not only considered plausible but formally confirmed, grounded in the established criminodynamic profile and the documented behavioural and psychological features of the perpetrator.

In conclusion, this case underscores the importance of an integrated medico-legal and forensic psychiatric approach in the investigation of fatal FDIA. While post-mortem findings alone may be insufficient to conclusively distinguish natural from non-natural asphyxial deaths in infancy, their interpretation within a broader clinical, behavioural, and psychosocial context can be decisive. Similarly, forensic psychiatric evaluation, when grounded in a solid factual and judicial framework, provides critical insights into the mechanisms underlying abusive behaviour without overstepping its clinical boundaries. The combined analysis of victim-centred autoptic data and perpetrator-centred psychiatric assessment thus represents a particularly robust model for the evaluation of complex and rare forms of fatal child abuse.

## Key points

1. Factitious Disorder Imposed on Another (FDIA) is a rare and severe form of child abuse, most often perpetrated by mothers.
2. This case involves a father who repeatedly induced respiratory crises in his three-month-old daughter, resulting in her death.
3. Medico-legal autopsy revealed non-specific findings of acute respiratory failure, excluding natural causes.
4. In the Italian legal framework, FDIA does not constitute a mental disorder that excludes or reduces criminal responsibility. The psychiatric assessment recognized that the defendant was fully responsible.
